# A Strainer-Based Platform for the Collection and Immunolabeling of Mouse Intestinal Organoids

**DOI:** 10.3390/ijms241713568

**Published:** 2023-09-01

**Authors:** Jinlong Tan, Yinju Liu, Weike Li, Guohua Chen, Yongxiang Fang, Xiaobing He, Baoquan Fu, Zhizhong Jing

**Affiliations:** State Key Laboratory for Animal Disease Control and Prevention, Key Laboratory of Veterinary Public Health of Agriculture Ministry Lanzhou Veterinary Research Institute, Chinese Academy of Agricultural Sciences, Lanzhou 730046, China; tjlong14@163.com (J.T.); liuyinju2022@163.com (Y.L.); liweike@caas.cn (W.L.); chenguohua02@caas.cn (G.C.); fangyongxiang@caas.cn (Y.F.); hexiaobing@caas.cn (X.H.)

**Keywords:** organoid, collection, labeling, 3D imaging, strainer platform

## Abstract

Intestinal organoids have emerged as powerful model systems for studying the complex structure and function of the intestine. However, there is a lack of widely applicable methods for the collection, labeling, and imaging of intestinal organoids. In this study, we developed a novel method for loading and labeling intestinal organoids, a method that efficiently collects the organoids and facilitates imaging of their three-dimensional (3D) structure. Based on this strainer platform, mouse intestinal organoids were adequately collected and immobilized, facilitating the immunolabeling workflow to target proteins of the organoids. After evaluation, the strainer size of 40 μm was considered to be more conducive to the collection and labeling of mouse intestinal organoids. More extensive research on organoids of multiple types and species origins will contribute to broadening the applicability of the methodology. Overall, our study proposes an innovative workflow for loading and analyzing intestinal organoids. The combination of a strainer-based collection method, fluorescent labeling, and 3D reconstruction provides valuable insights into the organization and complexity of these tissue models, thereby offering new avenues for investigating intestinal development, disease modeling, and drug discovery.

## 1. Introduction

Researchers have accumulated an abundant body of knowledge concerning the challenges posed by different diseases [1,2,3,4], including viral infections, cancers, metabolic disorders, and neurodegenerative diseases. However, there remains deficiencies in both cell and animal research methodologies. Cell research normally focuses on one or several cellular components [5,6] and, thus, cannot well describe biological processes occurring in vivo. Despite animal research being able to reproduce various disease processes [7,8], issues such as long experimental periods [9], animal mortality [10], ethics [11], and economics [12] remain problematic.

However, progress has been made due to the emergence of organoids [13,14]. Compared to cell research, organoids provide a similar microenvironment to target tissues and can accurately reflect biological processes in vivo [15]. In contrast to animal research, organoids significantly reduce the need for animal sacrifice, have lower research costs, and enhance experimental convenience [16]. In fact, organoids have been studied for decades but have only recently been defined using the term organoids, previously being known as three-dimensional (3D) tissue cultures [17]. These studies on organoids have numerous challenges, including the presence of partial rather than complete component cells, the lack of a vasculature or nervous system [18], and the absence of immune cells [19]. Furthermore, processes such as organoid collection, imaging, and adhesion need to be further optimized [20,21]. The traditional methodology for organoid collection and imaging relies on low-speed centrifugation followed by freezing, embedding, and sectioning [22]. These procedures, especially the freezing and sectioning, are of particular concern due to potential methodological limitations. Freezing may alter the tissue structure, and sectioning restricts observations of 3D organoids to two-dimensional (2D) images [23]. These limitations of 2D imaging of organoids raise several concerns, including how to represent the 3D characteristics of organoids and how to represent the spatial distributions and spatial interactions of proteins.

Recent evidence indicates that organoids can be imaged in 3D under multiple types of microscopes [24], suggesting that existing hardware devices are capable of reconstructing organoids in 3D. However, significant challenges exist in the process of organoid cleaning and immunolabeling. Traditional cell cleaning and immunolabeling procedures are typically performed using cell culture plates or dishes [25,26]. Cells generally adhere firmly to the bottoms of these plates or dishes, and this facilitates the cleaning and immunolabeling processes. However, organoids are embedded in matrix gels and need to have excess gel removed before immunolabeling [24], often making it impossible for the organoids to adhere to the plate or dish bottoms. If organoids are immunolabeled after removing the gel, repeated washing and reagent addition with a pipette will remove a large number of organoids, leaving only a minimal amount or even completely removing the organoids from the plate or dish bottoms. Therefore, it is necessary to utilize appropriate carriers to immobilize the organoids for immunolabeling and 3D imaging. One emerging approach is to culture organoids on high-throughput chips; this allows real-time monitoring of their development [27]. However, the technical challenges associated with optical components and laser beam-steering units can be daunting for researchers. Alternative simplified methods may provide more convenient conditions for 3D imaging of organoids.

Here, we provide a more convenient method based on the use of a strainer for collection, labeling, and 3D reconstruction of organoids. This study provides novel insights for organoid methodological research, contributing to fundamental research and drug development activities that employ organoids in metabolic disorders, neurodegenerative diseases, and neoplastic diseases.

## 2. Results

### 2.1. Overview of the Collection, Immunolabeling, and Three-Dimensional (3D) Imaging Protocols

**Figure 1:**
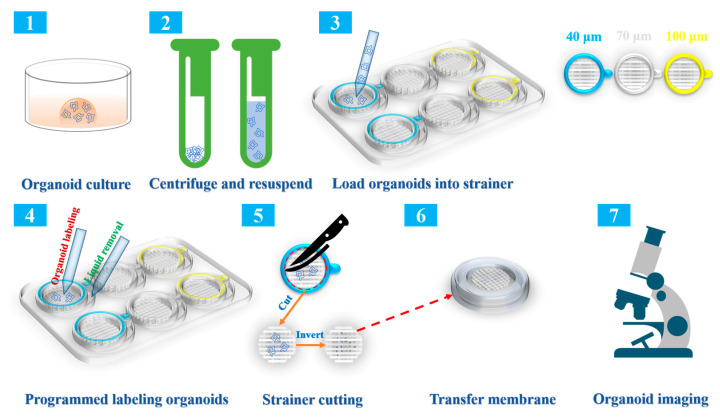
An illustration of the protocol for programmed labeling of organoids based on a strainer platform. Well-developed mouse intestinal organoids were collected with centrifugation in the bottom of a 15 mL centrifuge tube. After completely removing the matrix gel, the intestinal organoids were suspended and placed in the center of strainers of three sizes, namely, 40 μm (the blue strainer), 70 μm (the white strainer), and 100 μm (the yellow strainer). Subsequently, programmed labeling of the intestinal organoids was conducted. Liquid addition and removal were performed inside and outside the strainer, respectively. After labeling, the inner membranes of the strainers were cut and inverted into a glass bottom cell culture dish containing PBS for observation under a Nikon ECLIPSE Ti fluorescence microscope or a Leica SP8 confocal laser scanning device.

To visualize our approach, Figure 1 provides an illustration showing an overview of the protocol. As shown in the graphic overview, we first needed to culture intestinal organoids for the subsequent steps. Next, the Matrigel was removed through resuspending with pre-chilled PBS, centrifuging, and liquid removal. Subsequently, the mature organoids were resuspended again and loaded onto strainers of different sizes for immunolabeling. The organoids were subsequently immunolabeled according to standard procedures. The addition of a reagent was carried out using the strainer, while the removal of the liquid was performed in the gap between the strainer and the six-well plate; this prevented the organoids from being lost. For observing and 3D imaging of the organoids under the microscope, the strainer inner membrane was excised and inverted onto the bottom of a glass bottom cell culture dish containing PBS. For a more detailed explanation of the experimental procedures, see the Section 4.

### 2.2. Development of Small Intestinal Organoids in the Mouse

**Figure 2:**
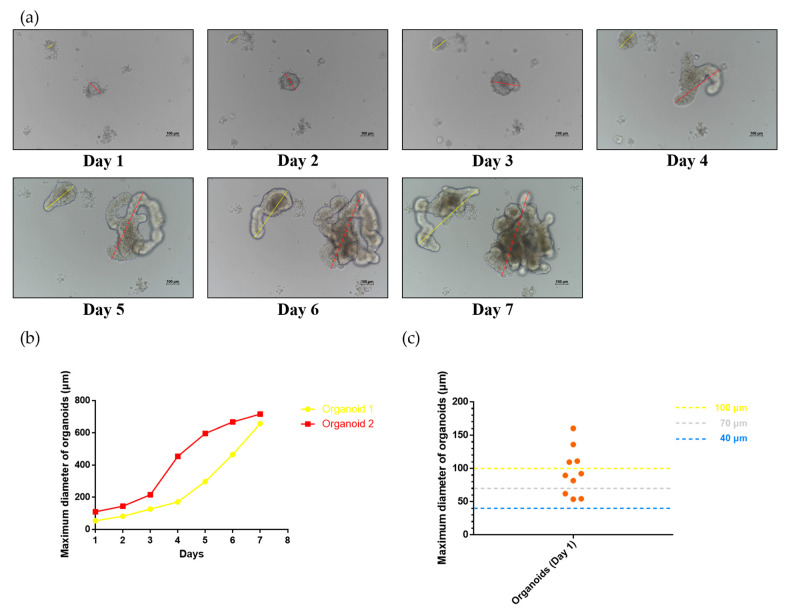
Development of mouse intestinal organoids. (**a**,**b**) The growth of intestinal organoids was observed daily (scale bar = 100 μm), and their maximum diameter was recorded (from day 1 to day 7). The yellow line indicates organoids in the upper left corner of the visual field. The red line indicates organoids in the middle of the visual field; (**c**) 10 organoids (the orange dots) were randomly selected, and their initial maximum diameters on day 1 were recorded. The dotted blue line indicates 40 μm strainers, the dotted white line indicates 70 μm strainers, and the dotted yellow line indicates 100 μm strainers.

A three-month-old Kunming (KM) mouse was euthanized with cervical dislocation to obtain the intestinal organoids. The small intestine was sampled, truncated, digested, and finally placed in a matrix gel in a three-dimensional manner. The crypts derived from niche stem cells were cultured in a 24-well plate supplemented with IntestiCult™ Organoid Growth Medium to develop into mature intestinal organoids. The morphology of intestinal organoids was monitored daily during culturing. Meanwhile, the maximum diameter of small intestinal organoids under the visual field was recorded daily. As shown in Figure 2a, it was possible to observe the developmental conditions of two intestinal organoids. One intestinal organoid located in the center of the visual field exhibited a budding phenotype on day 3, and this bud started to grow rapidly on day 4, eventually developing into a larger mature organoid by day 7. However, another intestinal organoid located in the upper left corner of the visual field showed the budding phenotype on day 5 and subsequently grew rapidly. Additionally, the maximum diameters of the two small intestinal organoids (marked by yellow and red lines in the figure) in the visual field were measured daily (Figure 2b). The results showed that the maximum diameters of the two small intestinal organoids increased with time. Moreover, 10 early organoids (day 1) were randomly selected, and their initial maximum diameters on day 1 were recorded. The initial diameters of all the organoids on day 1 were greater than 40 μm; the size of most organoids was greater than 70 μm, and only a few organoids were larger than 100 μm (Figure 2c). This suggested that the small intestinal organoids of mice could not easily pass through a membrane with a small pore size, and thus we speculated as to whether strainers with different pore sizes could be used as carriers to screen and label the organoids. Each small intestinal organoid growing in the matrix gel was unique concerning its phenotype based on morphology and size, factors that increase the uncertainty of the strainer as an organoid carrier.

### 2.3. Construction of a Strainer Platform and Programmed Labeling of Organoids

**Figure 3:**
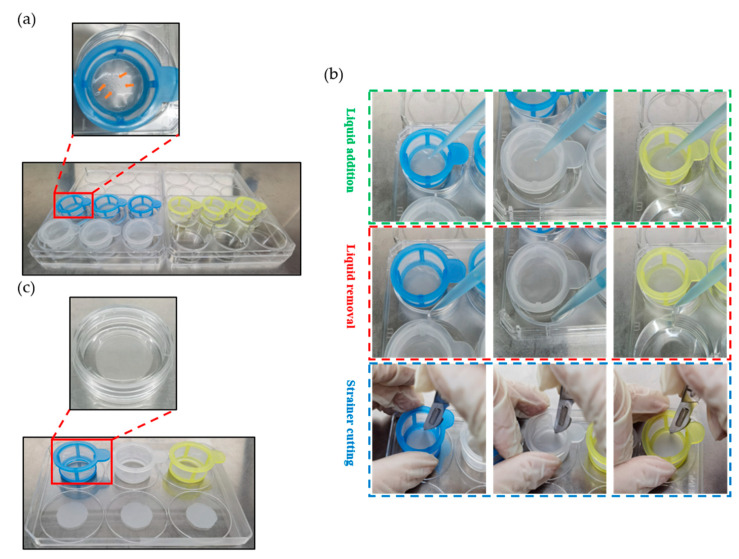
Practical operation of programmed labeling of organoids on the strainer platform. (**a**) Intestinal organoids were collected, resuspended, and added to the center of the strainer membrane. The visual field in the red frame was enlarged. The orange arrows indicate the loaded intestinal organoids; (**b**) intestinal organoids were program labeled. The dotted green frame indicates the procedure of liquid addition. The dotted red frame indicates the procedure of liquid removal. The dotted blue frame indicates the procedure of strainer cutting; (**c**) strainer membrane was cut and transferred into a glass bottom cell culture dish. The visual field of the dish in the red frame was highlighted.

As described in the Section 4, the strainer platform was constructed to load and label the organoids. In detail, the intestinal organoids were cultured in matrix gels for seven days. After culturing, the organoids underwent matrix gel removal, centrifugation, and collection. The organoids were subsequently transferred and loaded into strainers with different sizes. As shown in Figure 3a, the organoids were gently added drop by drop to the center of the strainer membrane. In the figure, the strainer is highlighted by the red box. Notably, we could observe that multiple intestinal organoids were present in the center of the strainer membrane. When complete loading of all the organoids on the strainers with different sizes was finished, the liquid addition procedure was performed inside the strainers (Figure 3b, green frame), and the liquid removal procedure was performed outside the strainers (Figure 3b, red frame) for labeling the organoids. After labeling, the inner membranes of the strainers with different sizes were sliced with a scalpel (Figure 3b, blue frame). Finally, as shown in Figure 3c (red frame), the sliced strainer membranes were transferred to a glass bottom cell culture dish containing PBS. The organoids were observed and imaged under a fluorescence microscope or a confocal microscope.

### 2.4. Evaluation of the Strainer Platform

**Figure 4:**
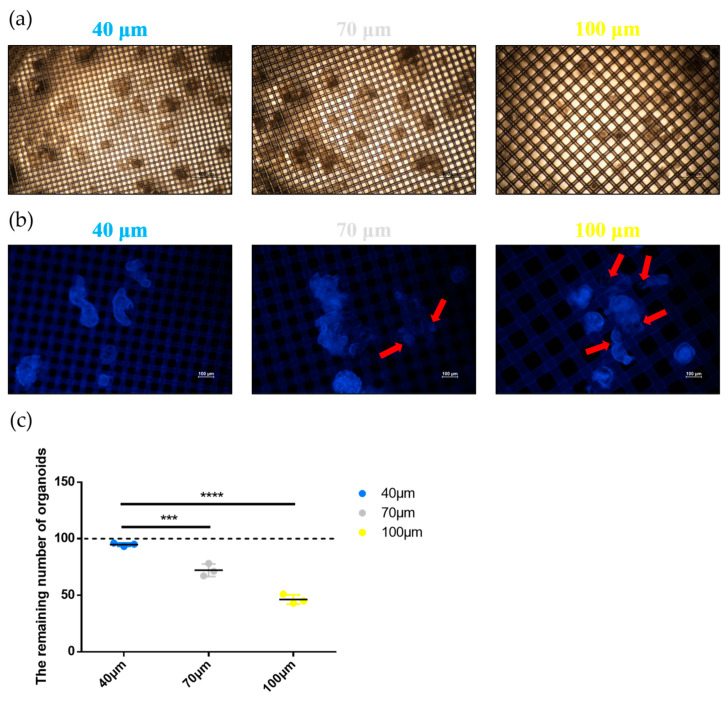
Evaluation of the strainer platform. (**a**) Strainer membranes were cut and placed in glass bottom cell culture dishes containing PBS without being inverted. Subsequently, intestinal organoids were observed under a Nikon ECLIPSE Ti fluorescence microscope in bright-field. Blue, white, and yellow indicate strainers of 40, 70, and 100 μm, respectively (scale bar = 500 μm); (**b**) Strainer membranes were cut and inverted in the glass bottom cell culture dishes containing PBS. The nuclei of the organoids were labeled using DAPI, and the organoids were observed under a Nikon ECLIPSE Ti fluorescence microscope. Blue, white, and yellow indicate strainers of 40, 70, and 100 μm, respectively (scale bar = 100 μm). The red arrows indicate the organoids are crossing the strainer membrane; (**c**) Number of remaining organoids was recorded. Blue, white, and yellow circles indicate strainers of 40, 70, and 100 μm, respectively. Asterisk indicate significant differences (***, *p* < 0.001; ****, *p* < 0.0001). The dashed line indicate the number of original organoids.

To evaluate the effect of the strainer platform on organoid collection, we observed the loading condition of organoids in strainers with different sizes. First, the strainer membrane containing the organoids was placed in a glass bottom cell culture dish containing PBS. The results (Figure 4a) showed that organoids were well attached to the surface of the strainer membrane. However, we found that the organoids were on the opposite side of the membrane, which prevented the organoids from being viewed under the microscope. Hence, we inverted the strainer membrane in the subsequent procedures for observing and imaging the organoids. During inverting, gentle manipulation is necessary because the process may cause organoid damage. To further determine the attachment status of the intestinal organoids on the strainer membrane, we labeled the nuclei of the organoids with DAPI. Co-labeling could emphasize the attachment of intestinal organoids to the membrane, as we found in our previous pre-experiments that DAPI labeled the nuclei as well as the strainer membrane, although the staining targeting the strainer membrane was nonspecific. We found that intestinal organoids loaded on the 40 μm strainer were well attached to the strainer membrane. However, the intestinal organoids loaded on the 70 μm strainer partially crossed the strainer membrane (red arrows). Interestingly, the phenomenon of organoids passing through the 100 μm strainer membrane (red arrows) was even more prominent (Figure 4b). These results indicated that a 40 μm strainer was best suited to loading the intestinal organoids, as the organoids on the two other sizes of strainers partially or even completely passed through the strainer membranes, and this could have altered the morphology of the organoids and was not conducive to observation under the microscope. Additionally, we calculated the remaining number (approximately 100 organoids were initially loaded onto each strainer) of intestinal organoids on the strainer membranes with different sizes. The number of intestinal organoids remaining on the 40 μm strainer membranes exceeded 90, which was significantly higher than on the 70 μm and 100 μm strainer membranes (Figure 4c). These results suggested that the 40 μm strainer was more effective for loading and labeling mouse intestinal organoids.

### 2.5. 3D Imaging of Small Intestine Organoids

**Figure 5:**
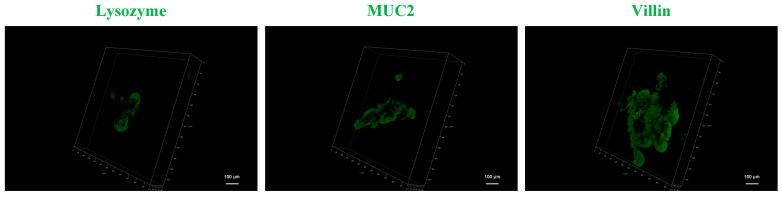
The intestinal organoids were labeled using anti-Lysozyme, anti-MUC2, and anti-Villin antibodies. The three-dimensional (3D) imaging of intestinal organoids was realized using *Z*-axis scanning from bottom to top under a Leica SP8 confocal laser scanning device (scale bar = 100 μm).

The distinct sizes of the strainers determined the remaining number and attachment status of the organoids on the strainer membranes. Based on the larger number and the more complete morphological characteristics of the organoids, the 40 μm strainer was used for 3D modeling. In brief, the intestinal organoids were program labeled as described above. Three marker proteins (Lysozyme, MUC2, and Villin) were used for labeling along with Goat Anti-Rabbit IgG H&L (Alexa Fluor^®^ 488) as the secondary antibody. The 3D structures of the mouse intestinal organoids labeled with Lysozyme, MUC2, and Villin were visualized under a Leica SP8 confocal laser scanning device (Figure 5). Video data for 3D organoid imaging are provided in the Supplementary Materials Videos S1–S3 (Video S1, Lysozyme; Video S2, MUC2; Video S3, Villin). The results highlighted the 3D distributions of these proteins in the organoids, thereby providing new insights into organoid 3D imaging methodology. The 3D reconstruction and protein distribution characterization of intestinal organoid marker proteins verified the feasibility of the strainer platform method, which provided a basis for the subsequent application of this platform to the 3D reconstruction and spatial distribution of other target proteins.

## 3. Discussion

Organoid research involves multiple tissues and organs, including the brain, fat, liver, and intestine [28,29,30,31]. Early organoid studies focused on single organoids without the presence of other cellular components or other organoid components [5], and such studies lacked an accurate reproduction of the organoid microenvironment. With the in-depth exploration of organoid research, multiorganoid and multicellular component research can better simulate and reproduce the organoid microenvironment [32], thereby providing sufficient support for multitype organ modeling and clinical application.

With the deepening of organoid research, technological innovations such as organoid imaging are essential. Currently, organoid imaging based on high-throughput chips plays a pivotal role in 3D representation [33], while difficulties in the preparation of fine electronic components narrow the scope of application of this method. Here, we investigated a novel approach concerning whether a strainer could assist in loading developed intestinal organoids for labeling and imaging. Notably, a strainer is commonly used to isolate intestinal niche cells. As indicated in our results, the maximum diameters of intestinal organoids on day 1 were distributed from tens of micrometers to more than 100 μm, suggesting that strainers with sizes 40 μm, 70 μm, and 100 μm could be used to attach the organoids. While strainers of all three sizes enabled organoids to attach to the membrane, only the 40 μm strainers preserved the majority of the organoids, as fewer passed through the strainer membrane. The evidence indicated that the 40 μm strainer contributed to organoid collection, labeling, and 3D modeling. While sufficient results were provided for confirming the usage of strainers with specific sizes, single mice used here may raise concerns about individual differences. However, the 6-well plates used here, compared to the use of 12-well plates or 24-well plates in traditional cell labeling procedures [34,35], greatly increased the efficiency of reagent use, especially the relatively expensive antibodies. Reducing the diameter of the strainer and employing a lower-depth six-well plate are straightforward ways to address the above concerns. The reduction in strainer diameter allows the labeling procedure to be performed on 12-well plates or even 24-well plates. The lower depth of the six-well plates allows the lower liquid level to cover the small intestinal organoids attached to the strainer membrane. Both methods can reduce the use of reagents and save research costs.

The applicability of the strainer platform to more types of organoids should be explored. Moreover, organoids of the same tissue derived from different species such as humans, rats, mice, swine, and monkeys may differ in methodology, and these differences may be due to the unique physical characteristics of organoids of different hosts [36]. The 3D structural differences as well as the size and development rate of organoids [37] may play key roles in the suitability of the strainer platform. The exploration of more organoids derived from species and distinct organ components in this platform can expand the application scope of this methodology. In summary, the recommended choice for mouse intestinal organoids is a commercialized 40 μm strainer. However, this size may not be suitable for smaller organoids when collecting and immunolabeling. At present, nonspecific staining caused by strainer materials is acceptable until new strainer materials are developed. In addition, for organoid immune labeling, imaging observation without removal of gels may be achieved in type I collagen gels [38]. Compared with the strainer platform, direct immune labeling of type I collagen gels may remove a large number of organoids during execution. However, it is undeniable that both of these labeling strategies are feasible.

Taken together, the methodology based on a strainer simplifies the procedures of collection, labeling, and 3D reconstruction in mouse intestinal organoids. The platform can also function in living organoid imaging. The establishment of the platform is expected to provide technical support for organoid-based research in drug development, tumor immunity, and neurological diseases.

## 4. Materials and Methods

### 4.1. Organoid Harvest and Culture

Mouse intestinal organoids were obtained from a Kunming (KM) mouse following a protocol for small intestinal organoid digestion. The small intestine was harvested after euthanizing the mouse. Specifically, a segment of the small intestine located 20 cm below the stomach was isolated by separating it from the surrounding mesentery, adipose tissue, and blood vessels. The segment was then thoroughly rinsed with pre-cooled PBS. After rinsing, the intestine was cut into 2 mm sections and collected in a 15 mL centrifuge tube. The intestinal segments were gently washed three times using 10 mL of pre-cooled PBS. Once the segments had settled naturally, the supernatant was removed, and this step was repeated 20 times until the supernatant became clear. Once the washing process of the intestinal segments was completed, the liquid was discarded, and the segments were suspended in 25 mL of Gentle Cell Dissociation Reagent (GCDR, 07174, STEMCELL Technologies, Vancouver, BC, Canada). The suspension was incubated at room temperature on a shaker set at 20 rpm for 15 min. The supernatant was removed after the intestinal segments had settled naturally. The segments were then gently resuspended three times in 10 mL of PBS containing 0.1% BSA by pipetting up and down. Once the suspension was complete, the supernatant was immediately sucked out and filtered using a 70 μm strainer (258368, NEST, Wuxi, China). The steps of resuspension and filtering the fragment were then repeated four times. The obtained filtrate was centrifuged at 290× *g* for 5 min at 4 °C. Subsequently, the precipitate was resuspended in 10 mL of pre-cooled PBS. The suspension was centrifuged again at 200× *g* for 3 min, and the supernatant was discarded. The collected precipitate was resuspended in DMEM, and the concentration and number of organoids were calculated through adding 10 μL of resuspension to the cell culture plate. Subsequently, organoids were centrifuged again at 200× *g* for 3 min. DMEM was removed to obtain organoid precipitation. The organoids were resuspended with IntestiCult™ Organoid Growth Medium (Mouse) (06005, STEMCELL Technologies) and combined with Matrigel^®^ matrix (356231, Corning, New York, NY, USA) in a 1:1 ratio. After that, 50 μL of the suspension was seeded into pre-warmed 24-well plates and allowed to completely solidify by placing the plates in a 37 °C incubator for 10 min. Finally, 750 μL of IntestiCult™ Organoid Growth Medium was slowly added along the side walls of each well. The organoids were then cultured at 37 °C and 5% CO_2_, and the culture medium was replaced every three days.

All the animal experiments described above were approved by the Guidelines of Lanzhou Veterinary Research Institute (Chinese Academy of Agriculture Science) for Institutional Animal Care. All the experimental procedures were performed in accordance with the Good Animal Practice Requirements of the Animal Ethics Procedures and Guidelines of the People’s Republic of China.

### 4.2. Organoid Passage and Collection

The organoids were passaged after culturing for seven days. In detail, the intestinal organoids growing in the Matrigel were directly resuspended in GCDR at room temperature and transferred to a 15 mL centrifuge tube. The tube was then incubated on a shaker at 200 rpm for 10 min, followed by centrifugation at 290× *g* for 5 min to remove the supernatant. Next, the precipitate was resuspended in pre-chilled DMEM and centrifuged at 200× *g* for 5 min to dissolve the Matrigel. After removing the supernatant containing the Matrigel, the organoids were resuspended in an equal volume of IntestiCult™ Organoid Growth Medium and Matrigel^®^ matrix and plated into 24-well plates. After three stable passages, the organoids growing in the Matrigel were directly collected in a 15 mL centrifuge tube, resuspended in 10 mL pre-chilled PBS to dissolve the Matrigel and centrifuged at 200× *g* for 5 min to remove the Matrigel. The supernatant was discarded, and the organoids were resuspended in 1 mL of PBS. The organoid concentration was calculated, and the volume of PBS was adjusted accordingly to achieve the desired organoid concentration for subsequent experiments.

### 4.3. Strainer Loading of Organoids

To collect organoids and perform subsequent procedural labeling, strainers with pore sizes of 40 μm (258369, NEST), 70 μm (258368, NSET), and 100 μm (258367, NEST) were utilized to accommodate the organoids, since their dimensions would align with the membrane pore size of the strainers. In detail, prior to the operation, the pipette tip was pre-wetted with PBS to prevent organoids from sticking to tube walls. The organoids collected in a 15 mL centrifuge tube were resuspended in PBS and added drop by drop to the center of the membrane, with approximately 100 organoids per strainer.

### 4.4. Programmed Labeling of Organoids

The intestinal organoids were collected and loaded onto strainers that were then placed on six-well plates. The intestinal organoids were program labeled using an indirect immunofluorescence assay (IFA) protocol. The primary antibodies involved in this research were Lysozyme (ab108508, Abcam, Cambridge, UK), Villin (ab97512, Abcam), and MUC2 (ab272692, Abcam). The secondary antibody was Goat Anti-Rabbit IgG H&L (Alexa Fluor^®^ 488) (ab150077, Abcam). In detail, the intestinal organoids were fixed with 4% paraformaldehyde (G1101, Servicebio, Wuhan, China) for 30 min and then washed three times with PBS. Next, organoids were permeated with 0.1% Triton X-100 (93443, Sigma, St. Louis, MO, USA) for 10 min and also washed three times with PBS. Subsequently, the organoids were blocked with 10% goat serum (SL038, Solarbio, Beijing, China) at room temperature for 30 min. After blocking, the liquid was directly removed, and then the primary antibodies were added, and the organoids were incubated at 4 °C overnight (dilution ratio of primary antibodies: Lysozyme = 1:250; Villin = 1:500; MUC2 = 1:500). After completing the primary antibody incubation, the organoids were washed three times with PBS. Then they were incubated at room temperature for 1 h with a secondary antibody (dilution ratio of secondary antibody: Alexa Fluor^®^ 488 = 1:500). After incubation, the organoids were washed three times with PBS. Finally, they were supplemented with PBS, and the samples were placed under a microscope for observation. Furthermore, while the nuclei were successfully labeled in 2D observation using DAPI (C0065, Solarbio), this staining was not employed in 3D labeling and imaging due to nonspecific staining observed with DAPI in the 2D observations.

### 4.5. Processing and Imaging of Organoids

After labeling the organoids, a surgical blade was utilized to make a circumferential incision along the inner membrane of the strainer. Subsequently, the detached strainer membrane was inverted and carefully positioned in either a cell culture dish or a confocal dish (801001, NEST) containing PBS for observation under a fluorescence microscope. For 3D imaging of the organoids, the Lysozyme, MUC2, and Villin-labeled intestinal organoids were scanned from bottom to top through the *Z*-axis under a Leica SP8 confocal laser scanning device for 3D modeling.

### 4.6. Statistical Analysis

Data were presented as mean and standard deviation (SD). The t-test was performed for the statistical analysis using GraphPad Prism software 6.01, and the *p* values represent significant differences (***, *p* < 0.001; ****, *p* < 0.0001).

## 5. Conclusions

The strainer platform plays a pivotal role in mouse intestinal organoid collection and immunolabeling. For mouse intestinal organoids, 40 μm is a suitable size to achieve these procedures and label target proteins. Given different types and sizes of organoids from other species or organs, the methodology based on the strainer platform remains different in various kinds of organoids, especially the size of the strainer. Accelerating the application of the strainer platform in organoids from other types and other species will confirm the feasibility of the methodology. Taken together, a strainer-based platform may contribute to the development of organoid research in the future.

## Data Availability

All data and materials are available from the corresponding author upon request.

## Supplementary Materials

The following supporting information can be downloaded at: https://www.mdpi.com/article/10.3390/ijms241713568/s1.
